# Sporotrichosis in renal transplant patients: two case reports and a review of the literature

**DOI:** 10.1186/s13256-020-02385-x

**Published:** 2020-06-26

**Authors:** Mazhar Hussein Amirali, Jacques Liebenberg, Sheylyn Pillay, Johan Nel

**Affiliations:** 1 Division of Nephrology, Department of Medicine, Faculty of Medicine and Health Sciences, Stellenbosch University and Tygerberg Hospital, Cape Town, South Africa; 2 Division of Medical Microbiology and Immunology, Department of Microbiology, Stellenbosch University and Tygerberg Hospital/National Health Laboratory Service, Cape Town, South Africa

**Keywords:** Case report, Renal transplant, Fungal infections, *Sporothrix schenckii*, Sporotrichosis

## Abstract

**Introduction:**

Sporotrichosis is a rare fungal infection in transplant patients; among these patients, it occurs mostly in renal transplant patients. *Sporothrix schenkii* is the primary pathogen responsible. A high index of suspicion is required to make the diagnosis keeping important differential diagnoses in mind. History of trauma through recreational or occupational exposure to the fungus may assist in making the diagnosis. Treatment is difficult, with long-term use of potentially nephrotoxic and cytochrome P450 inhibitor antifungal agents leading to potential calcineurin inhibitors toxicity. We describe two renal transplant patients presenting with distinct sporotrichosis infection: *“Case 2”* being only the second reported case ever of meningeal sporotrichosis. We subsequently review the general aspects of sporotrichosis, specifically in renal transplant patients as described in the medical literature.

**Case presentation:**

*Case 1*, a 43-year-old mixed ancestry male patient presented with a non-healing ulcer on the left arm for 1 year, he was diagnosed with cutaneous sporotrichosis and was successfully treated with itraconazole monotherapy. *Case 2*, a 56-year-old mixed ancestry male patient presented with a slow decline in functions, confusion, inappropriate behavior, rigors and significant loss of weight and appetite over the past 4 months, he was diagnosed with meningeal sporotrichosis and was successfully treated with a combination of deoxycholate amphotericin B and itraconazole.

**Conclusion:**

Physicians taking care of renal transplant patients should have a high index of suspicion for sporotrichosis infection particularly when conventional therapy for common conditions fails. Susceptibility testing is recommended to identify the most effective antifungal agent and its dose. The slow nature of growth of *Sporothrix schenkii* necessitates patients to be on amphotericin B until the time results are available. Finally, there is a need to be aware of potential drug-drug interactions of the azoles with calcineurin inhibitors and the required dose adjustments to prevent therapy related adverse events.

## Introduction

Sporotrichosis is a rare fungal infection in transplant patients and among these patients it occurs mostly in renal transplant patients. Extracutaneous forms of sporotrichosis – including meningeal sporotrichosis – without skin manifestations and no previous history of traumatic injuries have been described and are often difficult to diagnose. We present two renal transplant patients who presented with sporotrichosis, *case 1* with cutaneous sporotrichosis, the most common presentation, and *case 2* with meningeal sporotrichosis, which is exceedingly rare and is only the second case to have ever been reported in the literature. Both our patients were successfully treated with antifungal therapy which included itraconazole monotherapy for *case 1* and a combination of deoxycholate amphotericin B and itraconazole for *case 2*. We subsequently review the general aspects of sporotrichosis, specifically in renal transplant patients as described in the medical literature.

## Case 1

**Fig. 1:**
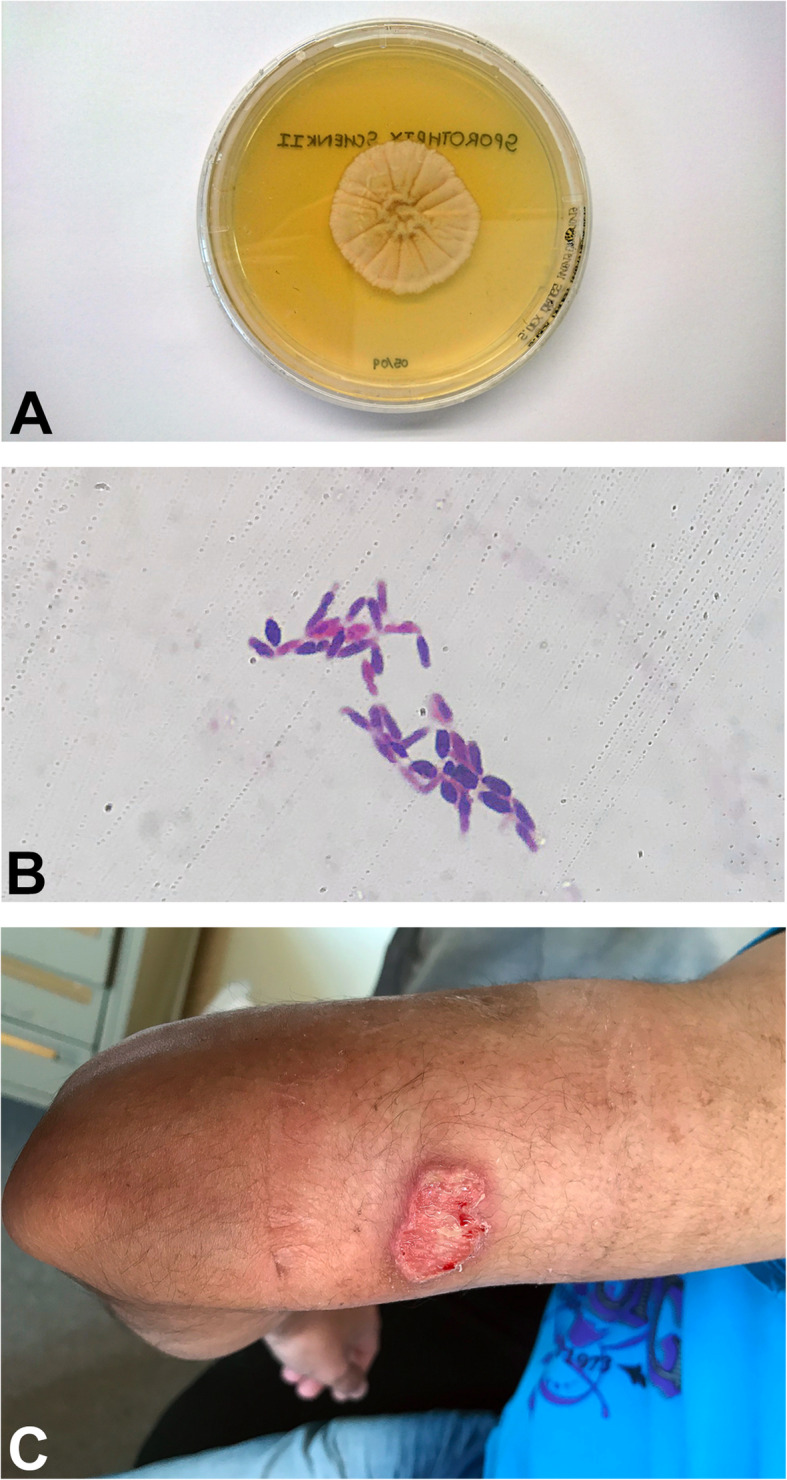
**a***Sporothrix schenkii* growth on Saboraud Dextrose agar – 28 °C. **b***Sporothrix schenkii* – typical “cigar” shaped budding yeast cells – 37 °C (Gram stain from cerebrospinal fluid (CSF), X 100 magnification). **a** and **b**: CSF specimen obtained from *case 2.***c** Left arm 2 X 3 cm non-healing ulcer with a granulated base and some purulent discharge (*case 1*)

A forty-three-year-old mixed ancestry male patient known to our division with end-stage kidney disease (ESKD) due to crescentic glomerulonephritis underwent a living related renal transplantation in 2009, with a current baseline creatinine of 118 μmol/L (64–104 μmol/L) and an estimated glomerular filtration rate (eGFR) of 58 mL/min/1,73 m^2^ (> 60 mL/min/1,73 m^2^). His immunosuppression regime consists of cyclosporine 150 mg twice daily, azathioprine 50 mg once daily, and prednisone 10 mg once daily. Other comorbidities include systemic hypertension, well controlled on amlodipine 5 mg once daily, and gastritis, well controlled with omeprazole 20 mg once daily. He presented in December 2018 with concerns of a non-healing ulcer on his left arm (Fig. 1c) for the past year which was nonresponsive to conservative therapy with daily saline dressings and empiric courses of antibiotics, including Amoxicillin. He had a history of being employed as a general worker in construction performing manual labour where he reports to have sustained injuries to his fingers and hands on multiple occasions. He has been working in the clothing factory for the past 6 years, he does not smoke or consume alcohol and does not give a history of gardening or other similar hobbies. The family and environmental history were unremarkable. Three years post-transplantation he presented with a non-healing ulcer of his left thumb which had developed from a nodule on the same digit. He received treatment in the form of surgical debridement and daily saline dressings which assisted with ulcer healing. He also reported features in keeping with nodular lymphangitis which resolved spontaneously prior to his current presentation. On physical examination he was clinically well, with a blood pressure of 116/71 mmHg, pulse rate of 72 beats/min and temperature of 36.0 °C. He had a 2 X 3 cm ulcer on the posteromedial aspect of his left arm which had a granulated base with some purulent discharge; his systemic examination was otherwise unremarkable. With concerns of malignancy post-transplantation, skin biopsies were performed. These revealed necrotizing granulomatosis with no malignant features. A repeat biopsy in March 2019 isolated *Sporothrix schenkii* on fungal culture and polymerase chain reaction (PCR) sequencing and he was subsequently initiated on oral itraconazole 400 mg once daily for four weeks followed by 200 mg once daily as life-long prophylaxis. His wound healed within 2 weeks of commencing treatment, he has been well since, and follows up regularly at our transplant clinic for review.

## Case 2

A fifty-six-year-old mixed ancestry male patient with end-stage kidney disease due to malignant hypertension was admitted in December 2018 on account of his family’s concerns of a slow decline in functions, confusion, inappropriate behavior, rigors and significant loss of weight and appetite over the past 4 months. He underwent a deceased donor renal transplantation in 2002 with a baseline creatinine of 91 μmol/L (64–104 μmol/L) and eGFR of 58 mL/min/1,73m^2^ (> 60 mL/min/1,73m^2^). His immunosuppressive regime consisted of tacrolimus 2 mg twice daily, prednisone 10 mg once daily, and mycophenolate mofetil (MMF) 500 mg twice daily. He used to work in the South African Police Services previously but has been unemployed for over 10 years, he does not smoke or consume alcohol and does not give a history of gardening or other similar hobbies. The family and environmental history were unremarkable. A review of his medical records revealed a remote history of articular sporotrichosis diagnosed on aspiration of his left wrist joint 2 years prior to his current presentation, for which he received treatment with oral itraconazole 200 mg daily for a total duration of 10 months. On physical examination he had a blood pressure of 135/72 mmHg, heart rate of 96 beats/minute, respiratory rate of 16 per/minute, temperature of 36.4 °C, random blood glucose of 12 mmol/L and, he appeared chronically ill with bilateral temporalis muscle wasting. His extracellular fluid compartment was contracted, and he had chronic non-pitting oedema of the lower limbs. He had symmetrical synovial hypertrophy on the small joints of his hands without evidence of a destructive arthropathy. Importantly, he did not have any stigmata suggestive of cutaneous or lymphocutaneous sporotrichosis. His sensorium was altered with a Glasgow Coma Scale of 12/15 (eyes – 4, motor – 5, verbal – 3), neither meningism nor focal neurological deficits were evident on the motor and sensory examinations. Examination of his fundi revealed no evidence of papilloedema. The rest of his physical examination was unremarkable. His work-up revealed patchy alveolar infiltrates on chest radiography. Computerized tomography of the brain was essentially normal apart from mild global cerebral atrophy and microangiopathic changes. Considering the history, clinical presentation, laboratory findings (Table 1) and the high background prevalence of tuberculosis in our population, as well as his immunosuppressed state, we decided to initiate empiric rifampicin sparing oral anti-tuberculous therapy (moxifloxacin 400 mg once daily, isoniazid 300 mg once daily, ethambutol 400 mg once daily, pyridoxine 25 mg once daily and pyrazinamide 1,2 g once daily). The patient’s clinical state continued to deteriorate despite being on anti-tuberculous treatment for more than 4 weeks. The difficulty was distinguishing a slow response to anti-tuberculous therapy in a patient with significant comorbidities from a misdiagnosis. Based on his deteriorating clinical state, previous remote history of articular sporotrichosis, and after reviewing the literature, we entertained the diagnosis of possible meningeal sporotrichosis and subsequently requested investigations specific for sporotrichosis which was confirmed by PCR in February 2019. Based on these results (Table 2) we discontinued his anti-tuberculous treatment and MMF and initiated intravenous deoxycholate amphotericin B (AMB-d) (0,75 mg/kg/day – adjusted to eGFR) which was continued for 35 days. Liposomal amphotericin B is prohibitively expensive in our setting, we therefore opted to treat him with AMB-d. Following this intensive phase of daily AMB-d, he showed a slow but favorable response to therapy (Table 3) and returned to a level of functioning that allowed independent out-patient living. On discharge, the dosing frequency of AMB-d was decreased to once weekly, given for a total of 4 doses, due to concerns of AMB-d related nephrotoxicity. He was also initiated on oral itraconazole 200 mg twice daily, which he was to continue for the next 12 months followed by 200 mg once daily as life-long prophylaxis. He had been stable and well at the time of this write-up; he unfortunately passed away at home, while asleep 3 months after discharge, an autopsy was declined by the family; the cause of death was presumed to be of cardiovascular nature.

**Table 1:**
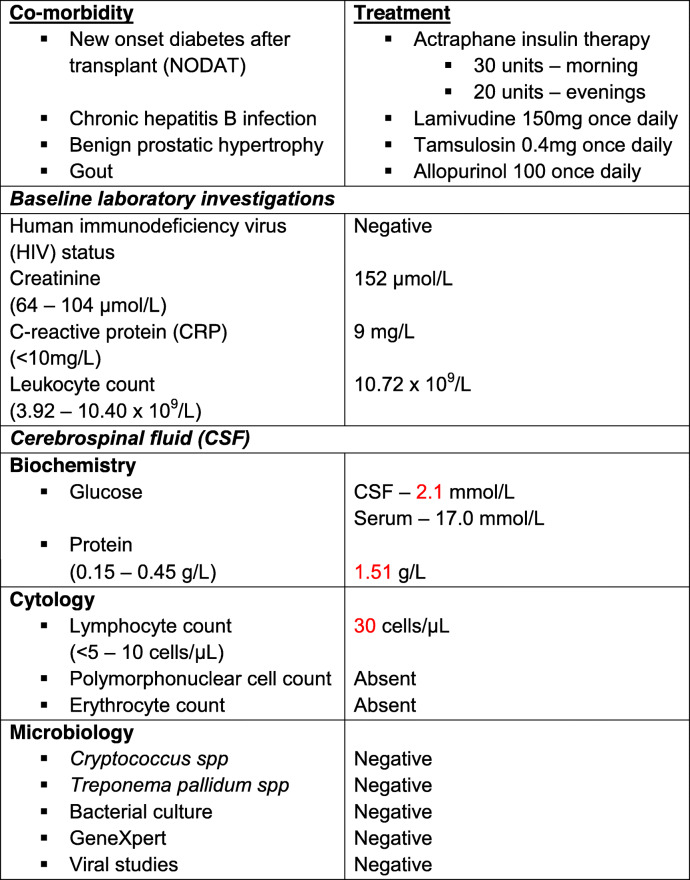
Clinical and laboratory data (*case 2*)

Red data signifies an abnormal value

*Spp* Species

**Table 2:**
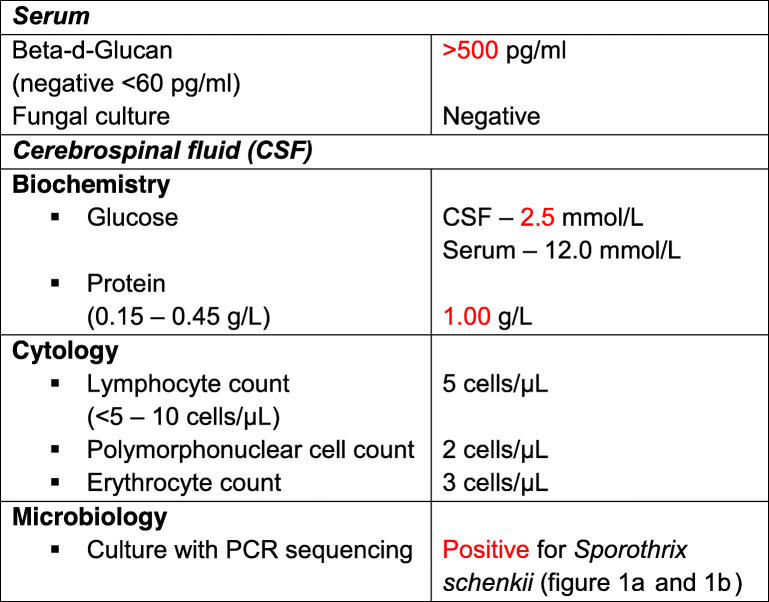
Specific laboratory testing for Sporotrichosis (*case 2*)

Red data signifies an abnormal value

*PCR* Polymerase Chain Reaction

**Table 3:**
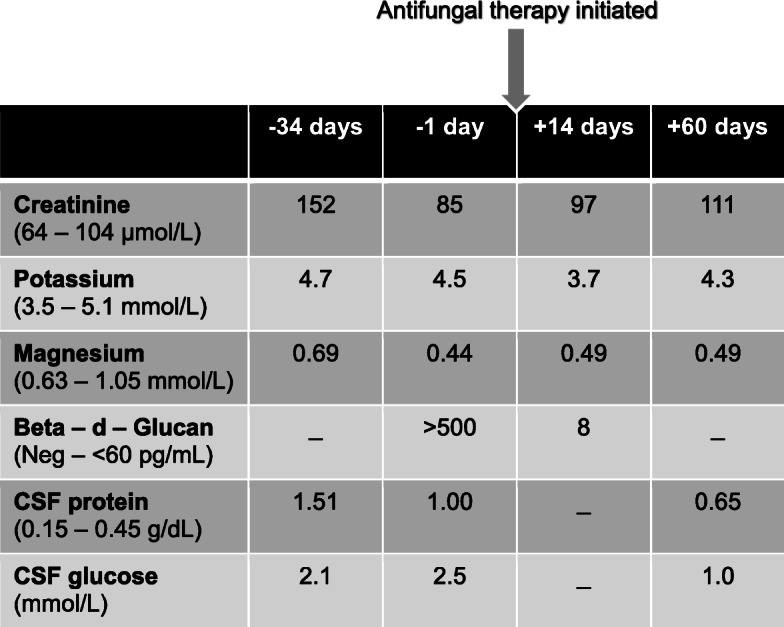
Serial laboratory values depicted in relation to initiation of antifungal therapy (*case 2*)

*Neg* Negative, *CSF* Cerebrospinal fluid

## Literature review

*Sporothrix schenkii* (SS) is a thermally dimorphic fungus – capable of existing as a saprophytic mold at environmental temperatures (25–30 °C) and a yeast at body temperature (37 °C) – belonging to the genus *Sporothrix*. SS is the primary pathogen responsible for sporotrichosis and spores of the mold are usually found in soil, damp or decaying wood and roses, and hence sometimes referred to as rose gardener’s disease. The fungus is usually inoculated through the skin and most frequently (95%) involves the cutaneous and subcutaneous tissues and lymphatics in immunocompetent patients. Persons at risk for disseminated SS are usually immunocompromised, and include, diabetics, those with chronic alcoholism, Human Immunodeficiency Virus/Acquired Immunodeficiency Syndrome (HIV/AIDS) and solid organ transplant patients. Zoonotic transmission from infected cats has been reported in Brazil and other South American countries where the disease is endemic [1]. The cutaneous form begins with a papule at the site of inoculation which may ulcerate or remain nodular with surrounding erythema. Drainage fluid may be odourless and non-purulent or pyogenic in nature, pain is usually mild with absent systemic symptoms. Similar lesions occur along the lymphatic drainage proximal to the index lesion, termed “Sporotrichoid” spread or nodular lymphangitis. Differential diagnoses include non-tuberculous mycobacterial infection, Nocardia and Leishmania [2, 3]. The disseminated form can involve various organ systems; including, pulmonary, osteoarticular, eyes (uveitis), and heart (endocarditis) [1]. Gullberg *et al.* [4] reported a recurring case of disseminated sporotrichosis in a renal transplant patient, involving joints, skin, and the central nervous system. This required three treatment cycles with systemic AMB-d. In the second cycle, intra-articular AMB-d was administered and in the third one, intrathecally. Immunosuppression was tapered to prevent further relapses. Agarwal *et al.* [5] reported a case of pyelonephritis due to SS in a renal transplant patient with a history of multiple renal calculi and recurrent urinary tract infections, the patient died in an accident before appropriate treatment could be initiated. Caroti *et al*. [6] reported SS infection in a renal transplant patient who initially presented with erythematous papulonodular lesion following trauma to the leg which responded to treatment with fluconazole. He presented again seven years later with acute osteomyelitis and gangrene in the same foot with ulcers. Biopsy of the ulcer revealed SS infection, MMF was discontinued and the patient responded well to fluconazole with regression of the lesions. Gewehr *et al.* [7] report two cases of sporotrichosis in renal transplant patients, the first patient had a prior history of trauma, presented with papular lesions on the hand and ear, while the second patient had no history of trauma, presented with generalized weakness, joint pain and disseminated nodular and ulcerated lesions involving the upper and lower extremities and the trunk. Both these patients were treated with itraconazole and amphotericin B. Table 4 summarizes cases of sporotrichosis in renal transplant patients reported in the literature to date.

**Table 4:** Summary of renal transplant patients with sporotrichosis (adapted from Gewehr *et al.* [7])

Author, Year	Age, years/ Gender	Type of renal transplant	Clinical manifestations (time after transplant)	History of trauma	Treatment	Outcome
Gullberg *et al.* [4], 1987	50/male	Deceased donor	Arthritis, meningitis (four years)	Unknown	AMB-d	Alive
Agarwal *et al.* [5], 1994	23/male	Living related donor	Pyelonephritis (nine months)	No	None	Death
Rao *et al.* [8], 2002	49/female	Deceased donor	Nasopharyngeal mass (six months)	No	None	Death
Caroti *et al.* [6], 2010	59/male	Unknown	Erythematous papular lesion on the left leg (unknown)	Yes	FLZ	Alive
P Gewehr *et al.* [7], 2013	48/female	Deceased donor	Plaques and ulcers on the hand and ear (nine months)	Yes	AMB-L; ITZ	Alive
	53/male	Deceased and living unrelated donors	Arthritis, draining and necrotic subcutaneous nodules in the limbs and trunk (one month)	No	AMB-L; ITZ; FLZ	Alive
This study	43/male	Living related donor	Non-healing ulcer - left arm (ten years)	Yes	ITZ	Alive
	54/male	Deceased donor	Pain and swelling – left wrist joint (fifteen years)	Unknown	ITZ	Alive
	56/male	Deceased donor	Constitutional symptoms, seizures, fluctuating mental state (seventeen years)	Unknown	AMB-d; ITZ	Death

*AMB-d* Amphotericin B deoxycholate, *AMB-L* Amphotericin lipid complex, *FLZ* Fluconazole, *ITZ* Itraconazole

## Discussion

Opportunistic infections are common in organ transplant patients, where fungal infections comprise 6% of all infections. Candida species, Aspergillus species and *Cryptococcus neoformans* are most frequently isolated [9]. Sporotrichosis is rare in transplant patients, and in these patients, it occurs mostly in renal transplant recipients, although Bahr *et al.* [10] reported sporotrichosis in a lung transplant patient and da Silva *et al*. [11] recently reported the first case of sporotrichosis in a liver transplant recipient. After a thorough search of the relevant literature our case (*case 2*) is only the second reported case of meningeal sporotrichosis in a renal transplant recipient, the first case was reported in 1987 by Gullberg *et al*. [4]. Our patient (*case 2*) differs from the one reported [4] by the way he presented clinically with features suggestive of tuberculous meningitis and no stigmata of cutaneous or lymphocutaneous sporotrichosis; thus making the diagnosis a challenge. Patients with meningeal involvement present with various symptoms, including: fever, headache, neck stiffness, vomiting, seizures, and altered mentation [1]. Important differential diagnoses include cryptococcal meningitis and tuberculous meningitis [12]. Investigations include a LP which is usually lymphocyte predominant with low CSF glucose and high protein levels. Tissue/CSF fungal culture is the gold standard and most sensitive method but may take longer to yield results while fungal PCR and sequencing can also be utilized to more rapidly identify SS [3]. Treatment is notoriously difficult given the paucity of clinical trials and the overlapping nature of the clinical features and laboratory investigations of this rare disease with more common aetiologies. Clinical practice guidelines of the Infectious Disease Society of America [13] recommend using itraconazole for cutaneous/lymphocutaneous involvement at 200 mg once daily for up to 2–4 weeks after the lesions have resolved – terbinafine 500 mg twice daily can be used as an alternative. Meningeal involvement is treated more aggressively with liposomal amphotericin B for at least 4 to 6 weeks at a dose of 3–5 mg/kg, followed by itraconazole 200 mg twice daily for at least 1 year. Deoxycholate (conventional) amphotericin B can be used as an alternative. Itraconazole prophylaxis at 200 mg once daily is to be continued life-long or until immunosuppression has been withdrawn. Calcineurin inhibitor dosage should be lowered by 33–66% to maintain the recommended therapeutic levels [14]. The significant inhibition of the Cytochrome P450 3A4 by the azoles leads to decreased metabolism with subsequent increased therapeutic levels of CNI’s leading to potential CNI toxicity. At our unit we reduce the total daily CNI dose by 66% following the first 24 hours of azole therapy, and subsequently adjust the dose according to the required through levels.

## Conclusion

Sporotrichosis is a rare opportunistic infection that occurs mostly in renal transplant patients; it can be localized (lympho-cutaneous) or involve various organ systems (disseminated). A high index of suspicion is required to make the diagnosis with the important differential diagnoses kept in mind. A history of trauma through recreational or occupational exposure to the fungus may assist in making a diagnosis. Treatment is difficult, with long-term use of potentially nephrotoxic and Cytochrome P450 3A4 inhibitor antifungal agents, which necessitate stringent monitoring of therapeutic drug levels to counteract CNI toxicity.

## Data Availability

Not applicable.

## Abbreviations

ESKD: end-stage kidney disease
eGFR: estimated glomerular filtration rate
PCR: polymerase chain reaction
MMF: mycophenolate mofetil
CSF: cerebrospinal fluid
AMB-d: deoxycholate amphotericin B
SS: *Sporothrix schenkii*
Spp: species
CNI: calcineurine inhibitor
LP: lumbar puncture
